# Aptness of *Escherichia coli* host strain CB390 to detect total coliphages in Colombia

**DOI:** 10.1038/s41598-019-45775-y

**Published:** 2019-06-25

**Authors:** Claudia Campos, Javier Méndez, Camilo Venegas, Luisa Fernanda Riaño, Paula Castaño, Natalia Leiton, Eliana Riaño

**Affiliations:** 1 Department of Microbiology. Faculty of Sciences, Pontificia Universidad Javeriana, Carrera 7 No. 40 – 62, Bogotá D.C., Colombia; 2 Department of Genetics, Microbiology and Statistics. Faculty of Biology, University of Barcelona, Av. Diagonal 643, 08028 Barcelona, Spain

**Keywords:** Bacteriology, Bacteriophages

## Abstract

Fecal bacteria have been used for more than a century as indicators of fecal contamination in water. In recent years, the monitoring of somatic and F-specific coliphages has been gradually included in guidelines and regulations as an additional parameter to reinforce water safety. The *Escherichia coli* host strain CB390 was tailored to detect both somatic and F-specific coliphages in a single test. The efficacy of this strain for bacteriophage detection, previously evaluated in Western Europe and North America, was assessed here for the first time in South America. The detection of somatic and F-specific coliphages by the strain CB390, as well as by standardized methods, was performed in drinking and river water and municipal and abattoir wastewaters. No statistical difference was found in the numbers of total coliphages detected by strain CB390 and the sum of somatic and F-specific coliphages determined separately by the standardized ISO methods. The data presented here provide further validation of the effectiveness of the host strain *E. coli* CB390 for the detection of total coliphages in waters in a single test and demonstrate its suitability for application in upper-middle income countries of the Americas (World Bank category).

## Introduction

The aptness of somatic and F-specific coliphages as indicators of viral and fecal contamination in water and food has been extensively studied, and the abundant scientific literature on this subject is covered in several reviews^1–5^. In the last few years, the monitoring of coliphages has been introduced in regulations or guidelines concerning the quality and management of waters for different uses^6–13^, as well as biosolids^14,15^ and food^16^. Relevant for the current study are the WHO guidelines about unplanned, unacknowledged or de facto potable water reuse^13^, which refers to the long-standing and common practice of producing drinking water from water sources impacted by wastewater discharges. Few water production systems worldwide, especially those using surface water, fall outside of these WHO recommendations.

Existing guidelines recommend testing for either somatic or F-specific coliphages or both, and standardized methods are available for both groups^17–20^. In most types of water samples, somatic outnumber F-specific coliphages^21^, whereas the opposite occurs in groundwater^22–24^ and reclaimed UV-irradiated water^25,26^. However, many of the aforementioned rules do not specify whether to test for somatic or F-specific coliphages, which has resulted in a lack of clarity and uncertainty about how to implement coliphage analysis.

In this state of affairs, it could be advisable to assess the numbers of both phage types, using either the standardized methods to detect somatic and F-specific coliphages separately or a bacterial host strain capable of measuring both in a single test. Ideally, the amount of phages detected by such a host strain should be identical to the sum of values obtained by the standardized methods. Guzmán *et al*.^27^ reported an *E. coli* strain CB390 tailored for the simultaneous detection of both somatic and F-specific coliphages, which functions as efficiently as the standardized ISO procedures (ISO 17050-1^17^ and ISO 10705-2^18^) for separate analysis. In subsequent tests, phage numbers detected by the strain were comparable with those of US EPA methods 1601^19^ and 1602^20^. Moreover, it has been successfully applied in different kinds of water samples in Spain and the USA^25,27–29^. In the current study, the suitability of CB390 to detect total coliphages was assessed in Colombia (South America), a country that differs from previously studied areas in income level (upper-medium according to the World Bank) and climate.

Thus, concentrations of *E. coli*, and somatic and F-specific coliphages detected by the *E. coli* strain CB390 were determined in an array of samples consisting of urban and abattoir wastewaters, river water and tap water. Considering the worldwide applicability of WHO guidelines for potable water reuse (13), the data provided here is relevant and useful.

## Materials and Methods

### Samples

One hundred and ninety-eight water samples with different levels of fecal contamination, representing diverse settings, were collected and analyzed over a 2-year period. Somatic and F-specific coliphages, bacteriophages infecting *E. coli* strain CB390 and *E. coli*, were analyzed from 48 samples of urban sewage, 84 samples of the Bogotá River collected in different sectors of the river with very different fecal contaminant loads, and 46 tap water samples of the Bogotá network. Regarding sewage samples, 18 of them were influent sewage of wastewater treatment plants and 30 were secondary effluents. Samples of drinking water were collected from faucets inside private homes throughout a marginal neighborhood, Ciudad Bolívar, occupied by citizens displaced by the political violence of the last decades. Inhabitants from this district normally use small homemade tanks for water storage and take water from taps that are fitted with gadgets to prevent water splashing. In addition, *E. coli*, somatic coliphages and bacteriophages infecting strain CB390 were analyzed in 20 samples of raw wastewater of abattoirs slaughtering porcine and bovine specimens.

### Sample processing

Before the phage analysis, wastewater, secondary effluents and river water samples were decontaminated by filtration through 0.22 µm diameter pore-size Millex-GP membranes, which are low-protein-binding polyethersulfone membranes (Millipore Corporation, Bedford, MA, USA).

The bacteriophages from samples corresponding to a section of the river with low fecal contamination and drinking water from the distribution network were concentrated from 1 liter samples in accordance with Méndez *et al*.^30^. Briefly, phages in 1000 mL of water samples were concentrated by adsorption to 0.22 µm pore-size cellulose ester membrane filters (GSWP; Millipore Corp., Bedford, MA), followed by elution with a solution of 1% beef extract, 0.05 M NaCl, and 3% Tween 80, and ultra-sonication.

### Bacteria enumeration

*E. coli* were enumerated by membrane filtration using Chromocult coliform agar (Merck, Darmstadt, Germany) supplemented with antibiotics (*E. coli*/coliform selective supplement; Merck). Dark-blue/purple colonies were presumed to be *E. coli* and confirmed by the addition of Kovac’s reagent^31^.

### Bacteriophage enumeration

Somatic coliphages were quantified by enumerating plaque forming units (PFUs) in the *E. coli* host strain WG5 according to the ISO 10705-2 standard method^18^. F-specific coliphages were quantified by enumerating PFUs in strain WG49 of *Salmonella enterica* serovar *typhimurium* according to the ISO 10705-1 standard method^17^. To quantify somatic and F-specific coliphages in a single assay, host strain CB390 was grown in MSB broth with 100 µg per ml of ampicillin. TYG agar and TGY semisolid agar^17^ supplemented with ampicillin (100 g ml^−1^), Ca^2+^, and Mg^2+^, as in the ISO 10705-2^18^ standard procedure, were used for the lower and upper layers in the double-agar-layer test, as indicated in Guzmán *et al*.^27^.

### Data treatment and statistics

The sum of the counts of somatic and F-specific coliphages was considered as total coliphages. To avoid dispersion due to the very different levels of fecal pollution of the tested waters, especially the river samples, the box-plots were calculated associating samples according to *E. coli* concentration intervals rather than by water origin.

Statistical analysis was carried out using R software package version 3.5.1^32^ and RStudio version 1.465^33^. Raw data were plotted as box-plots; the calculations performed to generate the box-plot graphs included quartiles and outlier values for each group of samples. Additionally, the data were plotted as quantile-quantile plots (qq-plots). Outlier values were determined according to Tukey’s method and were omitted from the statistical comparison tests.

Since not all data were normally distributed, the Wilcoxon rank sum test with continuity correction, the Kolmogorov-Smirnoff test and a two-sample Anderson-Darling test^34^ were carried out to compare the series of data. Whenever it is mentioned that differences were statistically significant or not, this is true for all three tests.

Values of coliphages and *E. coli* concentrations in the drinking water, where the positive detection frequency was very low, were estimated by applying Thomas’ formula for the calculation of the most probable number for long series of data^35^.

## Results and Discussion

**Figure 1:**
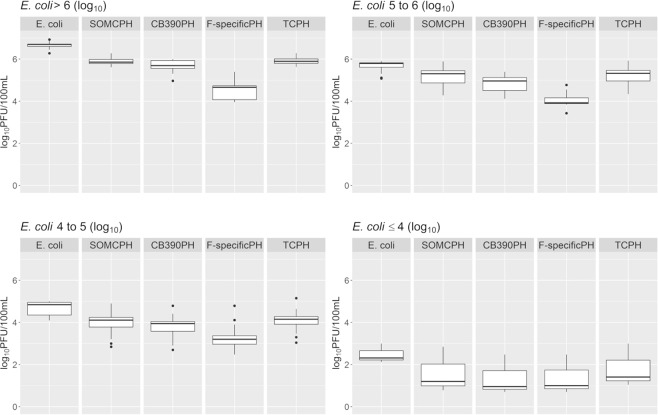
Boxplots of bacteriophage counts grouped by concentration of *E. coli* in the analyzed water samples. SOMCPH, CB390PH, F-specificPH and TCPH refer, respectively, to somatic coliphages, bacteriophages detected using the strain CB390, F-specific coliphages and total coliphages enumerated according to the ISO standard methods 10705-2 and 10705-1.

**Figure 2:**
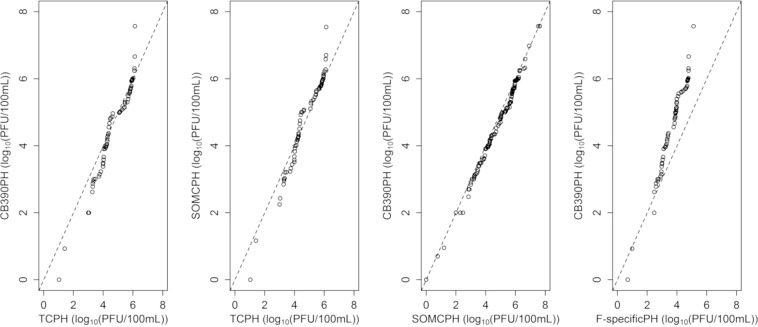
QQ-plot of bacteriophage counts. SOMCPH, CB390PH, F-specificPH and TCPH refer, respectively, to somatic coliphages, bacteriophages detected using the strain CB390, F-specific coliphages and total coliphages enumerated according to the ISO standard methods 10705-2 and 10705-1.

Figures 1 and 2 show the counts of the different indicators studied in urban and abattoir wastewaters and surface waters, indicating magnitude, order of abundance and the proportions among them, as described in the related literature^21^. These results are of interest, since there is a lack of comparative data for total coliphages detected by strain CB390 or other coliphages in the Americas.

The values obtained for drinking water are shown in Table 1. Worthy of mention, although out of the main scope of the current work, are the low values of fecal contamination found in drinking water samples, which is a positive result for a marginal neighborhood like the one studied here. The predominance of *E. coli* over the coliphages points to a source of contamination in the neighborhood, perhaps in the depots or due to cross-contamination, since water disinfection leads to higher proportions of coliphages than *E. coli*^36^. Unfortunately, the values of coliphages were too low to derive significant conclusions regarding the aptness of strain CB390 for counting total coliphages.

**Table 1:** Levels of *E. coli* and coliphages in drinking water. SOMCPH, CB390PH and F-specificPH state, respectively, for somatic coliphages, bacteriophages detected using the strain CB390 and F-specific coliphages.

	*E. coli*	SOMCPH	CB390PH	F-specificPH
Number of samples	44	36	44	44
% positive per litre	ND	0	4.5	6.6
% positive per 100 ml	13	ND	ND	ND
NMP per 100 ml	0.15	<0.0028	0.0041	0.0071

No statistical difference (*p* > 0.05) could be appreciated when the values of somatic coliphages, strain CB390 and the total coliphages were compared. Only the values of F-specific coliphages were significantly lower (*p* < 0.05) than the others. A comparison of the different bacteriophage counts in the waste and surface waters, sample-by-sample, is visualized in Fig. 2, as in other studies on somatic and F-specific coliphages in similar waters^5,21^. As described elsewhere^25,27,28^, the inclusion of F-specific phages to obtain total coliphages did not result in significantly higher values (*p* > 0.05) than those of somatic coliphages in the kinds of water tested. Additionally, the counts obtained by strain CB390, though slightly lower, are not significantly different (*p* > 0.05) from those obtained by the ISO procedure for somatic coliphages and total coliphages. This has also been observed in Europe^25,27^ and the USA when using US EPA methods to detect somatic and F-specific coliphages^28,29^. Therefore, these data reinforce the conclusion that the *E. coli* host strain CB390 is effective for the simultaneous detection of the total number of coliphages in different types of fecally contaminated water. Moreover, it is suitable for application in South American countries with upper-middle incomes.
